# Supplementary figures and images for: Mutational resilience of antiviral restriction favors primate TRIM5α in host-virus evolutionary arms races
Source: eLife. 2020 Sep 15;9:e59988. doi: 10.7554/eLife.59988 (PMC7492085; doi:10.7554/eLife.59988)

2/12/20

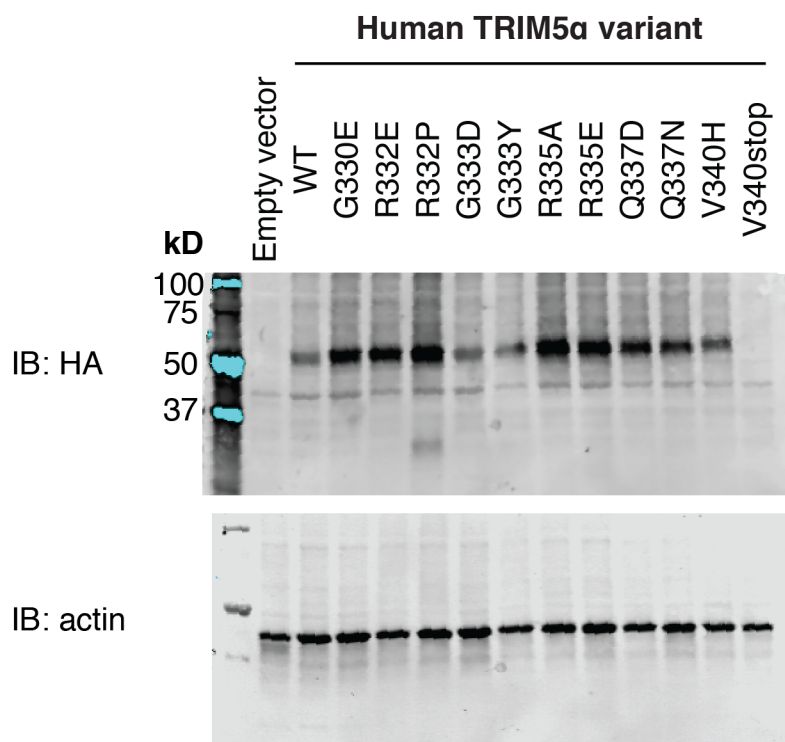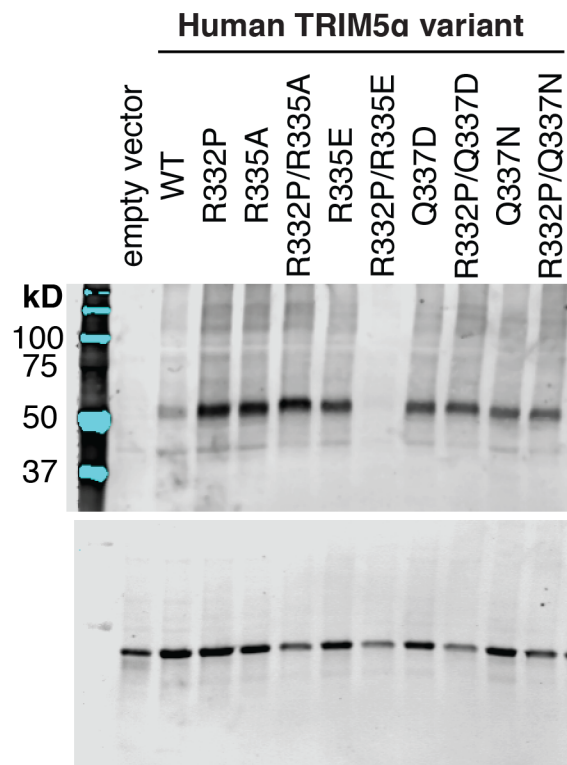

2/27/20

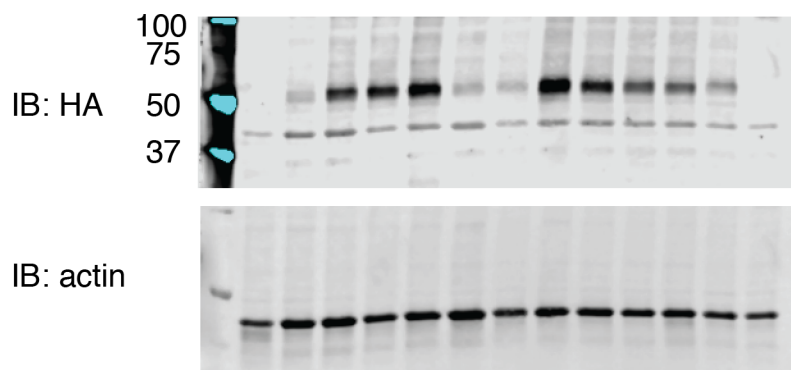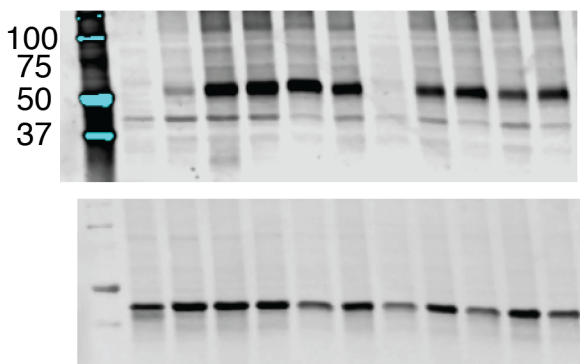

3/2/20

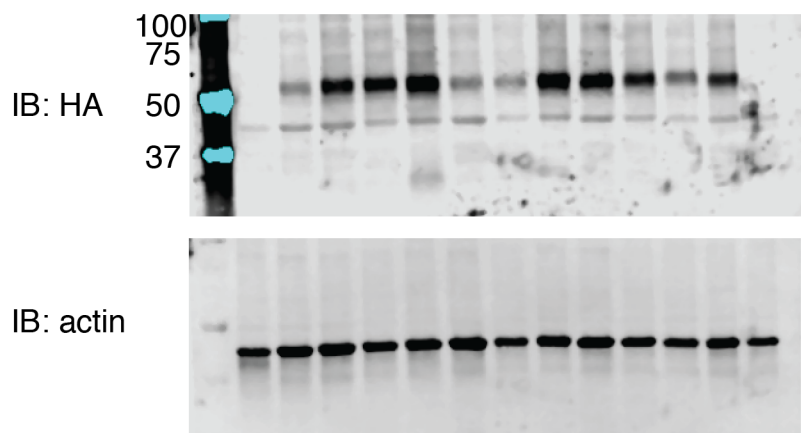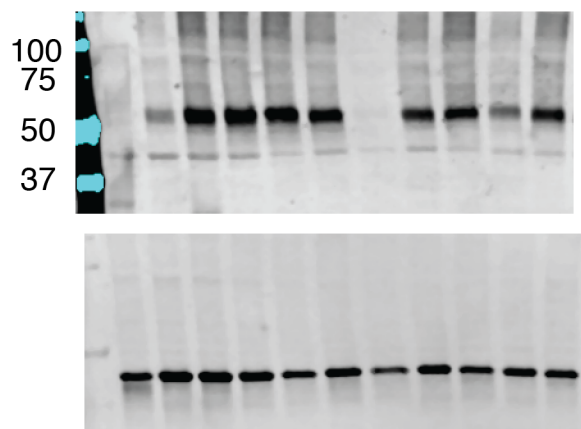

Supplement: Figure 3—source data 2. [file elife-59988-fig3-data2.png.pdf]

3/19/20

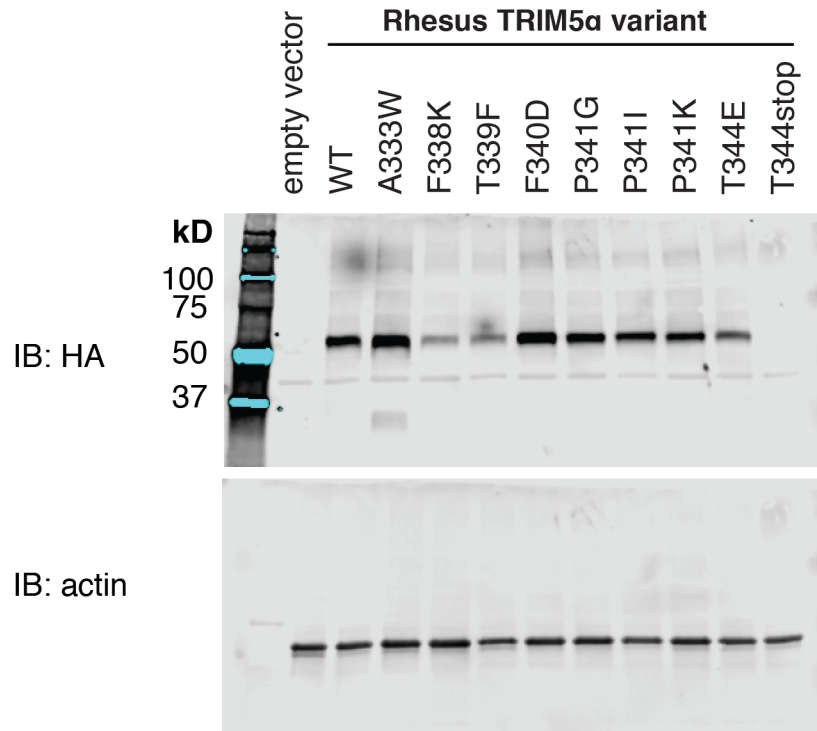

3/20/20

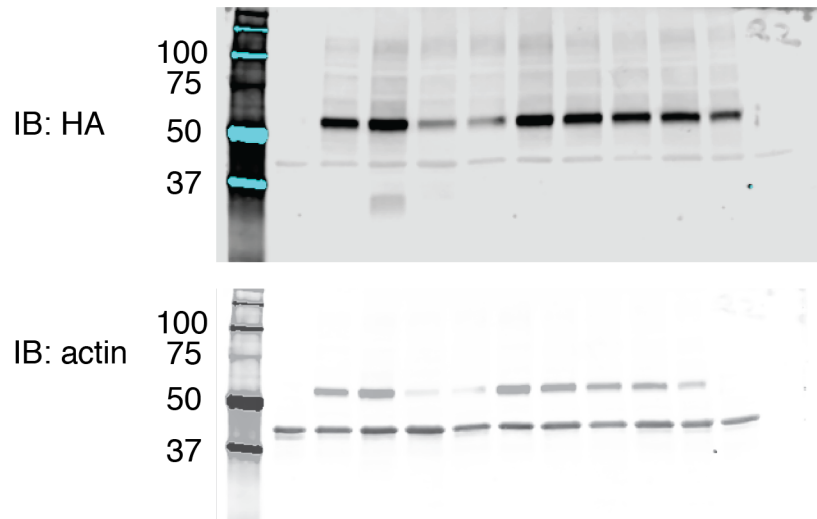

Supplement: Figure 5—source data 3. [file elife-59988-fig5-data3.png.pdf]
